# Experiences of Adolescents on Antiretroviral Therapy at Rustenburg Sub-District, Northwest Province, South Africa

**DOI:** 10.3390/children11020143

**Published:** 2024-01-24

**Authors:** Happy Maybe Maambiwa Khangale, Ndidzulafhi Selina Raliphaswa, Azwidihwi Rose Tshililo

**Affiliations:** Department of Advanced Nursing, Faculty of Health Sciences, University of Venda, Thohoyandou 0950, South Africa; 15001968@mvula.univen.ac.za (H.M.M.K.); rose.tshililo@univen.ac.za (A.R.T.)

**Keywords:** adolescent, antiretroviral, disclosure, experience, therapy

## Abstract

Background: Antiretroviral therapy (ART) is the treatment of people infected with human immunodeficiency virus (HIV) using anti-HIV drugs. The standard treatment consists of a combination of drugs (often called highly active antiretroviral therapy or HAART) that suppress HIV replication. As a result, people who have been infected live longer while on ART, which was initiated in South Africa in 2004. Aim: The study aimed to explore the experiences of adolescents on antiretroviral therapy in two primary health care clinics in Rustenburg sub-district Northwest Province. Methods: Qualitative, explorative, descriptive, and contextual approaches were adopted. Non-probability purposive sampling was used to select the healthcare facilities, and adolescents were chosen using convenience sampling. In-depth individual interviews were used to collect data from the participants. Only 13 adolescents between the ages of 15 and 19 years were interviewed. Collected data were analyzed using Tesch’s eight steps. Results: Qualitative themes identified included a description of the experiences of adolescents living HIV to adulthood and challenges experienced from childhood to adolescence period. Each theme had different sub-themes which included the paradoxical experiences of being diagnosed with HIV and being on antiretroviral treatment. Adolescents experienced poor adherence to ART due to treatment side effects such as drowsiness, change in body image, and headaches. Self-stigma resulted in adolescents not disclosing their status to their peers, closest and sexual partners which also made them not to adhere well in treatment. Conclusions: The study findings revealed that HIV-positive adolescents encounter various experiences while on ART, which causes them not to adhere to treatment. In the study, adolescents also experienced self-stigma which also affected their treatment adherence and brought fear of losing their loved ones.

## 1. Introduction

Antiretroviral therapy (ART) is an anti-HIV medication used to treat patients infected with the human immunodeficiency virus (HIV). The conventional treatment consists of a combination of medications that suppress HIV replication (commonly referred to as “Highly active antiretroviral therapy” or HAART) [1,2]. Antiretroviral medications have transformed the treatment of Human Immunodeficiency Virus (HIV) and acquired immunodeficiency syndrome (AIDS) since 1996, substantially improving the quality of life of individuals affected and prolonging the lives of many more. 

In 2016, an estimated 2.1 million (uncertainly bound 1.4–2.7 million) adolescents worldwide were living with HIV (ALHIVs) and on ART: 770 000 younger (aged 10–14 years) and 1.03 million older (aged 15–19 years) ALHIVs, with 84% living in Sub-Saharan Africa. South Africa (SA) accounts for 28% of the 80% of ALHIV cases in Sub-Saharan Africa [2]. 

One of South Africa’s most significant breakthroughs in the fight against AIDS has been the prevention of mother-to-child HIV transmission (PMTCT) program, which has lowered HIV transmission from mothers to their newborns from around 30% to 1.8% [3]. Thousands of babies, who were born with HIV before effective PMTCT was developed, are now struggling to understand what it means to be HIV positive, including why they are taking daily medication and dealing with the stigma of having a sexually transmitted virus despite not having been sexually active [3].

In South Africa, the availability of antiretroviral therapy, ART, has allowed children who were born with HIV to survive into their teens and beyond. In a similar vein, HIV sexual transmission is problematic due to rising incidence and prevalence among adolescents. [4] 

South Africa faces a period wherein children born with HIV survive into adolescence and beyond due to the provision of ART. Similarly, sexual transmission of HIV continues to pose a challenge as the incidence and prevalence among adolescents are increasing [4]; National Department of Health [5]. As a result, ALHIVs represent a sizable subgroup in the HIV and AIDS crisis that needs proper care.

ALHIVs are facing different experiences while they are on ART, which affect them every day. 

A total of 34 young individuals living with HIV participated in a Canadian study that revealed their ignorance and misperceptions regarding HIV treatment. Some of the participants said they had little say over their care, others mentioned disruptions to their lives, and still others said they had trouble adhering to ART. Adolescents’ adherence to ART may be influenced by a variety of factors, such as their reasons for seeking care, the severity of their disease, peer support, communication preferences during clinical interactions, structural problems, and their awareness of their HIV status [6,7].

The researcher found a few recent studies on the experiences of ALHIVs who are on ART in sub-Saharan Africa [8]. In particular, the researcher found few related studies conducted in SA. In a study conducted on SA adolescents with perinatal HIV/AIDS by authors et al. [9], six participants indicated that they did not know of their HIV-positive status while growing up; although they were taking medication, their caregivers did not disclose to them what it was for. This study focused on adolescents who are HIV-positive and are on ART.

The gap between adolescents’ experiences in other studies that covered the challenges and not experiences raised more questions and is of interest to the researcher as the study aims to explore the experiences of those adolescents between the ages of 15 and 19 years and who are on ART.

Therefore, it is in this light that the researcher found it to be beneficial to conduct this study to bring forth the experiences of ALHIVs and on ART since there are more changes every day in the medications and in the progress of HIV which affect adolescent’s day-to-day life. It would also be beneficial to the health care providers to be aware of what ALHIVs are experiencing while on treatment.

### 1.1. Problem Statement 

There is a rise in the number of virological failures among adolescents between the ages of 15 and 19 years in South Africa [10], which make it impossible to achieve the third 90 of the 90-90-90 strategy, which requires 90% of those on ART to be virally suppressed [11]. Adolescents on ART encounter different physical and social experiences, which may also contribute to the increase in virological failure. Studies have been carried out regarding HIV/AIDS in adolescents, mainly focusing on adolescents’ needs and challenges. However, individual personal experiences have not been explored and well documented, particularly in South Africa and throughout the rest of Africa [12]. Despite advances in survival, the childhoods of HIV-positive children and ALHIVs are often dominated by a plethora of complicated physical, psychological, and social stresses that damage their well-being and limit their potential to live happy, healthy, and full lives. In contrast to ART policy and programming, there has been very little commitment to the need for effective psychosocial therapies that can help people cope with their life experiences. Ref. [9] suggested that the issue of adolescents who are HIV-positive from birth is a problematic one. The study also showed that some parents raise their adolescents without disclosing their status, which is dangerous as it can cause unfavourable behaviours like denial when they find out. These adolescents may turn to rage and denial, which may negatively impact their future treatment compliance. In this regard, this is why semi structured interviews were conducted in order to explore the experiences of adolescents on ART.

### 1.2. Purpose of the Study

To understand the experiences of adolescents undergoing antiretroviral therapy in the Rustenburg sub-district of the Northwest province.

### 1.3. Research Method and Design

A qualitative, exploratory, and descriptive approach was used, to explore and describe the experiences of adolescents on ART. Two female researchers who are working in one of the South African Universities and one male researcher who is working as a nurse in one of the South African public clinics, sought a thorough grasp of the experiences of adolescents on ART as both researchers have research skills; hence, this approach was adopted as it allows the researcher to explore the lived experiences of adolescents on ART. 

### 1.4. Study Population

The study population consisted of adolescents aged 15–19 years living with HIV and are on ART in two selected primary health care facilities in Rustenburg sub-district Northwest province. The facilities were easily accessible since they are not far apart from each other and all of them had accessible adolescents on ART. The data of adolescents on ART were easily accessed from tier with the assistance of the facility data capturers.

### 1.5. Setting

The study was conducted in the Bojanala platinum District which is the largest of the four Districts of the North-West Province (Dr Kenneth Kaunda District Municipality, Dr Ruth Segomotsi Mopani District Municipality, and Ngaka Modiri Molema District Municipality). Moretele, Madibeng, Rustenburg, Kgetlengriver, and Moses Kotane are the four local municipalities that make up the Bojanala Platinum District. The Bojanala Platinum District accounts for a total population of 1.81 million of the total population in the Northwest province. Bojanala consists of 163 healthcare facilities.

## 2. Sampling Method

The health facilities and target population in this study were sampled using non-probability purposive sampling. The population was all adolescents on antiretroviral therapy between the ages of 15 and 19 years collecting treatment in the selected healthcare facilities. The qualifying adolescents were identified and recruited purposively for participation in the study during their clinic visits for collection of treatment. The sampling method was selected as it allowed for the selection of only ALHIVs and those who are on ART. A sample of (thirteen) 13 participants were interviewed from the two facilities which are HIV/AIDS primary health care facilities, of which six (6) participants were from Mfidikwe Clinic and seven (7) from Thekwane Clinic. Adolescents were recruited, and a good relationship was built. Those who consented to take part in the study were asked to sign a consent form after a full explanation of the study’s objectives and what participants should expect when they participate in the interviews. Moreover, those who were under 18 years old signed assent forms after their guardian or parents were consulted and gave consent for their children to participate in the study. Only the participant and the researcher were present in the interview room for privacy and confidentiality purposes as indicated in the Supplementary Materials.

### 2.1. Data Collection 

Data were collected from December 2020 to June 2021 using face to face in-depth individual interviews, which were audio recorded and transcribed after each interview. The pre-test was performed before the actual data collection to test the researcher’s probing skills and to detect any possible flaws that might occur during the actual data collection. Data collection was carried out by the first author in the participant’s local language. The interviews lasted for 30–45 min. Permission to use an audio recorder was obtained from the participants. The interviews were conducted in a consulting room with a well-closed door since it is good for participants’ safety and comfort in maintaining privacy. Saturation was reached with 13 adolescents when no new information emerged from the participants, which concluded the sample size. Measures to reduce the spread of COVID-19 were adhered to throughout data collection including hand sanitizer, 1.5 social distance, and wearing a mask. Field notes were documented during interviews. ALHIVs were asked two semi-structured questions, namely, when did you start taking ART? And what are your experiences while living with HIV and on ART? Adolescents were asked to freely express themselves about their experiences and respond to the follow-up probe questions asked by the researcher to gain more clarity which were dependent on the participant’s response. 

### 2.2. Data Analysis

Collected data were kept in a laptop and a voice recorder. The audio-recorded interviews were also transcribed. Each transcription was reviewed with the participants to verify and confirm that all the data were captured correctly before being shared with the supervisors, as well as the final layout of the themes, divisions, and subcategories. The data in this study were analyzed using Tesch’s open-coding approach [13]. The researcher and the independent coder then analyzed all transcriptions using line-by-line analysis. The primary researcher and supervisor discussed and came to a consensus and labeled contiguous parts of the text referring to comparable ideas (codes). *Step one*–By carefully reading all of the verbatim transcripts, researchers were able to obtain a sense of the overall picture. This provided suggestions for the data segments and how they appear/mean. The meaning evolved during the reading process, in which we wrote down all ideas as they came to mind. Researchers attentively and frequently studied and understood the transcripts of all participants. *Step two*—The collected phrases, sentences, and paragraphs were reduced and structured in a meaningful fashion to produce codes. Codes were built and adjusted as the coding process went on using open coding. 

*Step three*—A list of all the themes was prepared once the assignment for several subjects had been completed. Similar themes were grouped to make columns. The themes were divided into columns and categorized as significant topics, unique topics, and leftovers to make the process easier. To make the process easier, several colored pens were utilized. *Step four*—Topics are abbreviated to codes.

Topics that have evolved as codes have been abbreviated. These codes were written next to the appropriate transcription fragments. The codes were differentiated by containing all meaningful instances of a single code data. All of these codes were written in the margins of the page next to the data they represent, using a different pen colour from the one used in step three.

*Step five—The creation of themes and sub-themes.* The researcher created themes and sub-themes from coded data and associated texts and then decreased the entire list by combining issues that are related to one another to establish meaning for the themes and sub-themes. *Step six—Check for duplicate codes, subjects, and themes.*

In this step, the researcher starts over to remove any repetitions and, if needed, refine codes, subjects, and themes. Researchers looked for duplicate codes using the list of all codes. The codes that fit the description were recorded as needed and comparable codes were grouped. *Step seven—All themes and sub-themes are first grouped.*

After compiling all the collected data related to each theme into a single column and conducting a preliminary analysis, the researcher and co-coder met to decide on the themes and sub-themes that each of them had independently developed. 

### 2.3. Measures to Ensure Trustworthiness

Trustworthiness was ensured by using the four criteria, namely credibility, dependability, transferability, and conformability. Prolonged involvement established credibility by familiarizing himself with the issue and getting to know participants better by talking with them before the start of the interviews, this increased rapport and clarified descriptions with participants through familiarity. The researcher recorded the participants and transcribed the detailed data from Tswana to English. The audio and the detailed data were stored in a safe and secure place for confidentiality. For dependability, supervisors validated the methodology as they are experts in HIV-related matters, which was further enhanced using an independent coder to ensure consistency. 

Convenience sampling, thorough data collection, and comprehensive data descriptions, which include thorough accounts of participant background information, research context, and participant selection methodology, all contributed to ensuring the transferability of the results. Notes were kept in a safe in a safe place to guarantee conformance, enabling the establishment of a legitimate trail and the assessment of conclusions and interpretations if their sources could be located.

### 2.4. Ethical Consideration

The researcher sought permission from the following bodies to conduct the study: The Research Ethics Committee at the University of Venda. The research was presented to the School of Health Sciences Higher Degrees Committee, Ethics committee at the University of Venda, Sub-District and operational managers of primary health care facilities. Participants gave their permission voluntarily. Participants over 18 years signed consent forms whereas assent forms were signed by those who were under 18 years. Principles of beneficence, confidentiality, anonymity, and voluntary participation were adhered to, and participants’ information was not shared without their consent. Throughout the study, the researcher made sure that participants maintained their dignity. by ensuring that no one outside the research team had access to the raw data, confidentiality was guaranteed. The identities of the participants were kept secret.

## 3. Findings

Data were collected from 13 adolescents on antiretroviral therapy in Rustenburg sub-district Northwest selected primary health care facilities. Data from the participants were analyzed using Tesch steps and two major themes and their sub-themes emerged. Direct quotes from the participants were used to discuss and support each subject and theme. A summary of the findings is presented in Table 1 below:

**Theme 1:** **Description of the experiences of adolescents living with HIV to adulthood**
**Sub-theme 1.1:** **Paradoxical experiences of being diagnosed, living with HIV, and being on antiretroviral treatment explained.**

ALHIV and on ART are encountering different paradoxical experiences. As noted, six participants as displayed in Table 1 acquired HIV through sexual intercourse, which resulted in participants blaming themselves for not using condoms to protect themselves from being infected by HIV. The feeling of knowing that they could have prevented themselves from being HIV positive developed into anger towards self and disappointment.

Participant 4 said “*(taking a breath) Well, I was angry and disappointed at the same time, and I felt like it was the end of my life. I had a lot of questions in my head, wondering how and when I contracted the virus, and denial was creeping in mostly when I thought that the treatment was for life*.”

“*(looking down) When the test results came back positive for HIV, I knew it was the result of something I could have avoided if I had used a condom while having sex, but I didn’t, and I had to accept that I would have to take HIV treatment for the rest of my life*.” Participant 13.

**Sub-theme 1.2:** **Experiences related to adherence versus lack of adherence to ARTs while living with HIV.**

Adolescents in the study revealed different experiences related to adherence while living with HIV and on ARTs as shown in Table 2 below. The feeling of disappointment and the thought of thinking that one must take treatment became an emotionally stressful situation for participants. Adherence to treatments has been challenging from the time of treatment initiation as since treatment affects their daily routine, they had to adjust to side effects and the everyday routine of taking ARTs. Adhering to treatment was also affected by the lack of support in the household. This is supported by the participants as indicated below.

Participant 8 said “*Yes, I have missed treatment on a few occasions, particularly in the early days when there was a lot of bickering at home and no sign of being supported, which made me lose hope and believe it would be better if I died. I also miss treatment if I haven’t had enough sleep or if I am not at home*”.

**Theme 2:** **Challenges experienced from childhood to adolescence period.**

ALHIV experience different challenges transitioning from childhood to adolescence, this includes transitioning on ART with a poor understanding of why they are on treatment. Adolescents transitioning had the fear of stigma on how they will be treated if the community can be aware that they are living with HIV. The theme was divided into two sub-themes.

**Sub-theme 2.1:** **Poor understanding of the disease condition and lack of adherence to ARTs result from lack of disclosure by parents**

Table 1 above indicates that seven participants acquired HIV prenatally. Adolescents who acquire HIV prenatally grow up taking treatment not knowing exactly what it is for; some even transition from childhood still with no idea why they are taking treatment. Undisclosed HIV-positive status leads to adolescents not adhering to their treatment as they seek to know why they are on treatment while their peers are not. Adolescents placed more blame on their parents for not telling them about their status. Parents are responsible for the continuous partial disclosure of their children’s status as they grow up until they transition to the adolescent stage and give full disclosure to adolescents so that they can gain an understanding of their condition.

Participant 5 said “*I couldn’t take my treatment because I didn’t understand why, and the treatment I was taking was so hot that I couldn’t stomach swallowing it, so I didn’t take it every day. On days when I pretended to take the treatment but didn’t, I would act as if I did*”.

Participant 6 said, “*I also miss my treatment, especially when I recall that my mother never cared to inform me of my status*”.

Participant 10 said, “*Yes, I have missed several treatment doses, especially before I knew it was ART, when I used to spit off the treatment in the toilet, and I have also missed additional treatment doses when I was still struggling with denial*”.

**Sub-theme 2.2:** **fear of stigma and discrimination experienced.**

The study revealed that participants experienced feelings of fear, despair, and fear of stigma and discrimination. The self-stigma experienced by adolescents leads to them having negative beliefs about themselves. This included adolescents feeling ashamed of themselves, and experiencing low self-worth and low self-esteem which manifested as they battled denial of their HIV-positive status.

Participant 11 said “*(taking a breath) Well, I was angry and disappointed at the same time, feeling as if it was the end of my life. I had a lot of questions in my head, wondering how and when I contracted the virus, and denial was creeping in. I kept on fearing what people would say if they discovered my treatment, what would they say if they saw me taking ART at the clinic? My friends will leave me (looking down)*”.

Participant 9 explained, “*I feel better as each day goes by while taking treatment and I’m gaining strength and overcoming denial, even though life has changed, and I’m afraid of how people will react if they find out about my status and taking treatment*”.

Adolescents had the fear of community stigma and discrimination as they did not want the community, including their schoolmates, to know about their HIV-positive status. This made them worry that frequent visits to the clinics would bring suspicions. Therefore, adolescents preferred their parents to collect treatment on their behalf.

Participant 3 said “*Well, I had to live in fear that if my schoolmates found out, it would be the end of me. It makes it tough for me to talk about my problems with other people, especially the problem of my everyday experiences of taking ART, the side effects I encounter while on ART*”.

Participant 7 said, “*I’m also having trouble attending medical appointments; I’d rather have my mother collect the treatment for me so that my friends don’t interrogate me*”.

## 4. Discussion 

The study focused on the experiences of ALHIVs and those on ART. Descriptions of the experiences of ALHIVs and challenges experienced from childhood to adolescence emerged as major themes. For a description of the experiences of ALHIVs, the sub-themes were, “paradoxical experiences of living with HIV and being on ART, Experiences related to adherence versus lack of adherence to ARTs while living with HIV”. The sub-themes that emerged from challenges experienced from childhood to adolescence included ‘Poor understanding of the disease condition and lack of adherence to ARTs resulting from lack of disclosure by parents’, fear of stigma, and discrimination experienced.

The adolescents in the study who acquired HIV sexually revealed different experiences related to adherence while taking ARTs. The battle of wanting to know when and how one became infected with the virus was one of the experiences which made them not adhere well to treatment. Furthermore, they blamed themselves for not using protection against HIV while having sexual intercourse. The feeling of disappointment and the thought that one must take treatment became an emotionally stressful situation for participants as they knew undergoing treatment would interfere with their day-to-day life as it would be an everyday routine. 

Adherence to ARTs was challenging as they had to adjust to side effects and the everyday routine of taking ARTs. The findings are like those of the study carried out by [14]. ALHIVs have long complained about side effects of ART such as drowsiness, as well as physical changes like enlarged buttocks. Similarly, in a study carried out by [15,16], drug tiredness, inconvenient dose frequency, complex drug regimen, unwanted side effects, and clinic visits that coincide with school schedules are the most frequently stated impediments to effective adherence. They also fear that their love partners and suspicious peers might find out about the uninvited disclosure of their status. 

The researcher noted that healthcare providers should further educate adolescents on the side effects of treatment as they do have an impact on the adolescents’ adherence and their day-to-day life can be disturbed by the treatment side effects; this should be carried out continuously in every visit so that they feel supported and relieved from the thought of defaulting due to side effects. Healthcare workers should raise the issue of side effects to the adolescents and their caregivers so that the caregiver is able to support the adolescent while they are experiencing any side effects. Sexual partners should be indexed to test them and educate them on HIV so that they can support their partner even when they are experiencing side effects. Adolescents’ adherence clubs should be utilized by healthcare workers to educate them on side effects, for which they can even use posters during club meetings to display the side effects. 

In the study, adherence to treatment was also affected by non-disclosure of the HIV positive to the family by the adolescents. ALHIVs and those who acquired the disease peri-natal also expressed poor adherence to treatment due to poor disease knowledge. Disclosure of one’s HIV status was not given by parents, which led to children transitioning to the adolescent stage and starting to wonder why they are on treatment while their peers are not on treatment. Delay of disclosure of HIV-positive status also leads to adolescents neglecting ART and blaming parents for not disclosing the status. 

The lack of disclosure by parents led to a poor understanding of the disease condition and a lack of adherence by the adolescents in the study. The findings are like those of the study carried out by [17], which revealed that adolescents believed that it seemed unfair for them to monotonously take ART medication every day, especially when they felt fit, a practice common among adolescents whose seropositive status had not been disclosed. Such adolescents often skip drugs or completely quit medication if they are not closely monitored, which negatively impacts their medication adherence. Status disclosure, counselling, and informing adolescents about the potential side effects of the medication will help promote adherence to HAART.

In the study carried out by [18], adolescents in the study had been vertically infected with HIV and faced disclosure issues. Following the initiation of ARTs, children’s HIV + status was kept disguised, and they had no idea what the medicine was for. Non-disclosure of HIV status to HIV-infected children and adolescents, according to [19], is a known impediment to them adhering to ART. The study revealed that participants grew up and transitioned to adolescence without knowing what the treatment they were using was for. The lack of knowledge regarding why they are on treatment led to difficulty in adhering to treatment by adolescence. The researcher notes that disclosing HIV to adolescents at an early age promotes adherence. Continuous partial disclosure should be revealed by parents as children grow up, and when they transition to the adolescent stage a full disclosure should be given to the adolescents to improve adherence to ARTs.

The findings revealed that adolescent’s adherence to ART is affected by their wanting to know why they are not living a normal life like other adolescents who are not under ART. The fear of stigma also resulted in poor adherence to adolescents as they were afraid of what their friends would say when they were taking treatment. These findings are similar to those of [18], who discovered that adherence to ART by adolescents frequently deteriorates for a variety of reasons, including forgetting to take medications, stigma, body image issues, and a desire to be normal [19]. While they recognized the value of ART in their lives, two individuals in this study reported weariness and apathy with adherence to the treatment regimen. They also admitted that ART therapy interfered with their lifestyles at times. They have, nonetheless, never stopped taking them.

Ref. [20] defined self-stigmatization as a process in which a person becomes aware of negative stereotypes finds that they agree with those judgments and applies the stigmatized beliefs to themselves. Self-stigma was a common challenge that adolescents reported to be a barrier to disclosing their HIV status as well as seeking services. In the study, adolescents experienced feelings of self-worthlessness, hopelessness, and negative body image; these negative experiences were emphasized by the adolescents’ facing difficulties of disclosure and sexual relationship complications. This led to adolescents experiencing emotional distress as they feared that if their peers could find out about their status they would be judged. This resulted in adolescents missing their treatment doses and ending up with poor adherence. While experiencing poor adherence due to the emotional distress of self-stigma, adolescents still did not feel ready to disclose their status. 

This is further supported by the findings of the study, which revealed that adolescents felt that by disclosing their HIV status, they would not be accepted by their community. This realization caused stress and fear in trying to decide whether to disclose to friends or family. Several of the adolescents were still in school, a place that commonly facilitates gossip and social exclusion towards PLHIVs [21]. This caused most adolescents to avoid disclosing their HIV status to anyone. However, the burden of this secret made many of the adolescents feel shameful and stressed, leading them to avoid engagements with friends, skip doctor’s appointments, and stop taking their medication. This caused both physical and emotional health problems for the participants and likely explains the high rates of virological failure seen in this group [22,23]. The study carried out by [12] also emphasizes that younger and older adolescents aged 10–14 years and 15–19 years, respectively, were less likely to achieve virological suppression compared to adults in the final model.

## 5. Conclusions

The present study explored some of the experiences experienced by ALHIVs and those on ARTs. ALHIVs and those with acquired HIV blamed themselves for being HIV positive as they felt that they would have protected themselves from becoming infected with the virus. Poor adherence was common amongst the adolescents due to ART side effects and self-stigma. Delayed disclosure by parents to adolescents who acquired HIV perinatally also affected their adherence to ART as they wanted to know more about why they are on treatment and what the treatment is for. Continuous disclosure by parents would be beneficial to improving treatment adherence. ALHIVs fear that their peers and family will find out about their need to be on ART, which will result in them losing their loved ones and losing trust in them as they fear that they will be discriminated against due to their status. The study also revealed the need for more future research to be carried out on adolescents on ARTs. Further research will assist in achieving good adherence to treatment as adolescents will be able to share their experiences and receive individual support based on individual experiences. 

### 5.1. Limitations of the Study

The study was only conducted in the selected primary healthcare facilities of the Rustenburg sub-district in Northwest Province. Data were collected using the participant’s local language. However, no certified translation was carried out.

### 5.2. Recommendations

Adolescents on antiretroviral therapy encounter different experiences physically and socially which may also contribute to the increase in virological failure. Studies have been carried out regarding HIV/AIDS in adolescents, mainly focusing on adolescents’ needs and challenges. However, individual personal experiences have not been explored.

The nursing staff should continuously engage with the adolescents during their visits to the facilities to gain an understanding of the adolescent’s experiences while on ART; this will allow adolescents to feel supported and improve their ART adherence. Campaigns should also be conducted in communities to educate the public about ART, reducing the stigma directed at people on ART. The Department of Education should place a greater emphasis on HIV education in schools so that there is no HIV stigma in schools since students are unaware of the virus. Parents and guardians of HIV-positive adolescents should be taught more about the significance of disclosing their HIV+ status to their children rather than lying about it, which only decreases the adherence to ART. Future researchers should investigate more adolescents’ individual experiences while on ART among both perinatal and vertically infected adolescents.

## Figures and Tables

**Table 1:** Biographical information of participants.

Participant	Age	Gender	Type of Treatment	Level of Education	Socioeconomic Status	Perinatal Acquired HIV or Sexual Transmission
1	17	M	ART	Grade 12	Depending on social grant	Perinatal
2	18	M	ART	Grade 12	Depending on social grant	Sexual transmission
3	19	F	ART	Tertiary	Depending on social grant and Bursary	Sexual transmission
4	17	F	ART	Grade 11	Depending on parents income	Sexual transmission
5	18	F	ART	Grade 12	Depending on social grant	Perinatal
6	18	F	ART	Grade 11	Depending on granny grant	Perinatal
7	19	M	ART	Tertiary	Depending on parents income	Perinatal
8	18	F	ART	Grade 12	Depending on social grant	Perinatal
9	17	F	ART	Grade 11	Depending on parents income	Sexual transmission
10	18	M	ART	Grade 12	Depending on granny grant	Perinatal
11	17	F	ART	Grade 11	Depending on social grant	Perinatal
12	19	M	ART	Tertiary	Depending on Bursary	Sexual transmission
13	19	M	ART	Grade 12	Depending on social grant	Sexual transmission

**Table 2:** Themes and sub-themes.

Themes	Sub-Themes
**1.** **Description of the experiences of adolescents living with HIV to adulthood**	1.1 Paradoxical experiences of living with HIV and being on antiretroviral treatment explained. 1.2 Experiences related to adherence versus lack of adherence to ARTs while living with HIV.
**2.** **Challenges experienced from childhood to adolescence period**	2.1 Poor understanding of the disease condition and lack of adherence to ARTs resulting from lack of disclosure by parents. 2.2 fear of stigma and discrimination experienced.

## Data Availability

The data presented in this study are available on request from the corresponding author. The data are not publicly available due to the principle of confidentiality which was assured to the participants.

## Supplementary Materials

The following supporting information can be downloaded at: https://www.mdpi.com/article/10.3390/children11020143/s1. Consolidated criteria for reporting qualitative studies (COREQ): 32-item checklist. Ref. [24] is cited in Supplementary Materials.
