# Supplementary material for: Experiences of Adolescents on Antiretroviral Therapy at Rustenburg Sub-District, Northwest Province, South Africa
Source: Children (Basel). 2024 Jan 24;11(2):143. doi: 10.3390/children11020143 (PMC10887175; doi:10.3390/children11020143)
Supplement: Supplementary file 1 [file children-11-00143-s001.zip › children-2737874-supplementary.pdf]

Manuscript: Experiences of adolescents on antiretroviral therapy at Rustenburg sub-district, Northwest Province, South Africa

Consolidated criteria for reporting qualitative studies (COREQ): 32-item checklist

Developed from:
Tong A, Sainsbury P, Craig J. Consolidated criteria for reporting qualitative research (COREQ): a 32-item checklist for interviews and focus groups. *International Journal for Quality in Health Care*. 2007. Volume 19, Number 6: pp. 349 – 357

No. Item	Guide questions/description	Reported on Page #
**Domain 1: Research team and reflexivity**		
Personal Characteristics		
1. Inter viewer/facilitator	Which author/s conducted the interview or focus group?<br>All the authors	Page 4 line 147
2. Credentials	What were the researcher's credentials?<br>2 PhDs and 1 Master's in Nursing (Mcur)	Page 1
3. Occupation	What was their occupation at the time of the study?<br>2 lecturers and one nurse	Page 108 and 109
4. Gender	Was the researcher male or female?<br>Two females and one male	Page 108 and 109
5. Experience and training	What experience or training did the researcher have?<br>Research skills as a researcher	Page 110
Relationship with participants		
6. Relationship established	Was a relationship established prior to study commencement?<br>yes	Page 5 line 132<br>.
7. Participant knowledge of the interviewer	What did the participants know about the researcher? E.g. personal goals, reasons for doing the research	Page 4 line 133-134
8. Interviewer characteristics	What characteristics were reported about the inter viewer/facilitator? E.g. Bias, assumptions, reasons and interests in the research topic<br>Reasons for research topic	Page 4 line 133

**Domain 2: study design**		
Theoretical framework		
9. Methodological orientation and Theory	What methodological orientation was stated to underpin the study? E.g. grounded theory, discourse analysis, ethnography, phenomenology, content analysis<br>Qualitative study	Page 4 line 104-106
Participant selection		
10. Sampling	How were participants selected? E.g. purposive, convenience, consecutive,<br>Participants were selected purposively	Page 5 line 125 -126
11. Method of approach	How were participants approached? E.g. face-to-face, telephone, mail, email<br>Face-to-face	Page 6 line 142
12. Sample size	How many participants were in the study?<br>13 participants	Page 5 line 130 to 132
13. Non-participation	How many people refused to participate or dropped out? Reasons?<br>None	Page 4 line 173
Setting		
14. Setting of data collection	Where was the data collected? E.g. home, clinic, workplace	Page 5 line 131<br>.
15. Presence of non-participants	Was anyone else present besides the participants and researchers?<br>No	Page 5 line 137 and 138
16. Description of sample	What are the important characteristics of the sample? E.g. demographic data, date<br>Age	Page 5 line 126
Data collection		
17. Interview guide	Were questions, prompts, guides provided by the authors? Was it pilot-tested?<br>Yes, it was pre-tested and two semi-structured guiding questions used	Page 4 line 143 -144<br>And 150-152
18. Repeat interviews	Were repeat inter views carried out? If yes, how many?<br>No	N/A
19. Audio/visual recording	Did the research use audio or visual recording to collect the data?	Page 6 line 146

	Yes, audio-tape was used	
20. Field notes	Were field notes made during and/or after the inter view or focus group<br>Yes, field notes were done during interviews	Page 6 line 148
21. Duration	What was the duration of the inter views or focus group?<br>30 to 45 minutes	Page 6 line 143
22. Data saturation	Was data saturation discussed?<br>Yes	Page 4 line 146
23. Transcripts returned	Were transcripts returned to participants for comment and/or correction?<br>Yes, they were returned	Page 6 line 156-158
**Domain 3: analysis and findings**		
Data analysis		
24. Number of data coders	How many data coders coded the data?<br>One independent coder	Page 6 line 159
25. Description of the coding tree	Did authors provide a description of the coding tree?	N/A
26. Derivation of themes	Were themes identified in advance or derived from the data?<br>From the collected data	Page 7 line 184 and 185
27. Software	What software, if applicable, was used to manage the data?<br>Laptop and voice recorder	Page 6 line 156
28. Participant checking	Did participants provide feedback on the findings?<br>Yes	Page 4 line 168
Reporting		
29. Quotations presented	Were participant quotations presented to illustrate the themes/findings? Was each quotation identified? E.g. participant number<br>Yes, all done	Page 10 to 13 line 230 to 315
30. Data and findings consistent	Was there consistency between the data presented and the findings?<br>Yes, consistency is there	Page 8 to 10<br>Lie 217 to 230
31. Clarity of major themes	Were major themes clearly presented in the findings?<br>Yes, they were	Page 10 line 230 to 315
32. Clarity of minor themes	Is there a description of diverse cases or discussion of minor themes?	Page 10 to 13 line 230 to 315

	Yes, Discussion of major and minor themes were done	
